# Significant Benefits of Nanoparticles Containing a Necrosis Inhibitor on Mice Testicular Tissue Autografts Outcomes

**DOI:** 10.3390/ijms20235833

**Published:** 2019-11-20

**Authors:** Federico Del Vento, Maxime Vermeulen, Bernard Ucakar, Jonathan Poels, Anne des Rieux, Christine Wyns

**Affiliations:** 1 Gynecology-Andrology Unit, Medical School, Institute of Experimental and Clinical Research, Catholic University of Louvain, UCLouvain, 1200 Brussels, Belgium; federico.delvento@uclouvain.be (F.D.V.); vermeulen.maxime@live.be (M.V.); jonathan.poels@uclouvain.be (J.P.); 2 Advanced Drug Delivery and Biomaterials Unit, Louvain Drug Research Institute, Catholic University of Louvain, UCLouvain, 1200 Brussels, Belgium; bernard.ucakar@uclouvain.be (B.U.); anne.desrieux@uclouvain.be (A.d.R.); 3 Department of Gynecology-Andrology, Saint-Luc University Hospital, 1200 Brussels, Belgium

**Keywords:** necrosis inhibitor, nanoparticles, prepubertal testicular tissue, transplantation, fertility preservation, spermatogonia, prepubertal boys, necrosis, tissue engineering, male fertility

## Abstract

Fertility preservation for prepubertal boys relies exclusively on cryopreservation of immature testicular tissue (ITT) containing spermatogonia as the only cells with reproductive potential. Preclinical studies that used a nude mice model to evaluate the development of human transplanted ITT were characterized by important spermatogonial loss. We hypothesized that the encapsulation of testicular tissue in an alginate matrix supplemented with nanoparticles containing a necrosis inhibitor (NECINH-NPS) would improve tissue integrity and germ cells’ survival in grafts. We performed orthotopic autotransplantation of 1 mm³ testicular tissue fragments recovered form mice (aged 4–5 weeks). Fragments were either non-encapsulated, encapsulated in an alginate matrix, or encapsulated in an alginate matrix containing NECINH-NPs. Grafts were recovered 5- and 21-days post-transplantation. We evaluated tissue integrity (hematoxylin-eosin staining), germ cells survival (immunohistochemistry for promyelocytic leukemia zinc-finger, VASA, and protein-boule-like), apoptosis (immunohistochemistry for active-caspase 3), and lipid peroxidation (immunohistochemistry for malondialdehyde). NECINH-NPs significantly improved testicular tissue integrity and germ cells’ survival after 21 days. Oxidative stress was reduced after 5 days, regardless of nanoparticle incorporation. No effect on caspase-dependent apoptosis was observed. In conclusion, NECINH-NPs in an alginate matrix significantly improved tissue integrity and germ cells’ survival in grafts with the perspective of higher reproductive outcomes.

## 1. Introduction

During the last decades, the improvement of oncological therapies increased cancer survival rates for pediatric patients in Europe [1]. However, one potential side effect of chemotherapy and radiotherapy is a permanent loss of fertility [2]. For prepubertal boys facing such treatments, the only option for fertility preservation is cryobanking of immature testicular tissue (ITT) containing spermatogonia as the only cells with reproductive potential, with the perspective of future autologous use of banked tissue [3,4]. 

To respond to a concern that deeply compromises the quality of life after cancer, several clinics have initiated a fertility preservation program for the male pediatric population [5,6,7]. The procedure consists of testicular sampling and cryopreservation of small testicular fragments. The satisfaction of patients and parents has been surveyed, and the results showed that acceptance rates reached 74% when a multidisciplinary care pathway was organized [8].

To restore fertility once the patient is cured, several strategies using patient’s own frozen-thawed ITT have been proposed, such as autotransplantation of whole tissue pieces [9] or spermatogonial stem cells [10], in vitro maturation (for review see [11]), and testicular organoids generation for in vivo or in vitro use [12,13,14]. 

Based on ITT transplantation outcomes in several animal species (for review see, [15]) and on the very recent achievement of a live birth in non-human primates [16], transplantation of the cryostored testicular tissue back to the cured patient appears to be the most promising strategy to offer these patients a chance to father their own genetic child.

With regard to human ITT, xenotransplantation to castrated nude mice showed spermatogonia’s ability to survive, proliferate, and even differentiate up to the stage of pachytene spermatocyte [17], although complete spermatogenesis was not achieved. Besides an incomplete differentiation, all human ITT xenotransplantation experiments were characterized by a limited spermatogonial survival [17,18,19,20,21,22]. Hypoxia and oxidative stress were suggested to be responsible as the procedure of grafting is an avascular transplantation technique where no artificial anastomosis between the grafted fragment and the host vascular system is performed [9]. Such procedure exposes the grafted tissue to a period of hypoxia during the first 5 days (as demonstrated for xenotransplantation of ovarian tissue [23]), before its revascularization with neo-vessels sprouting from the grafted tissue and connecting with the vascular system of the host [24]. The consequent ischemia/reperfusion increases the production of radical oxygen species (ROS) [25,26] that might harm testicular tissue and cells. 

Considering the limited amounts of testicular tissue that can be cryostored for each patient and the scarcity of spermatogonia stem cells in the testis [27], our objective was to define a transplantation condition that would be the most efficient in terms of survival of spermatogonia. Several investigators have already assessed the impact of graft supplementation with various molecules or growth factors. Recent encouraging results were reported when 5 days in vitro culture step of human tissue with vascular endothelial growth-factor (VEGF) was performed before xenografting to the nude mice [22]. However, hormone supplementation with testosterone [21] or follicle-stimulating hormone (FSH) [20] and the use of antioxidant molecules [21] did not improve graft outcome. Hence, alternative approaches need to be investigated using both new molecules and alternative drug delivery strategies. 

Tissue engineering techniques that combine biomaterials to protect testicular tissue and nanomedicines, providing localized and sustained drug delivery have been considered [28], and privileging local controlled delivery are important in the context of cancer as molecules acting as survival factors on distant tumor sites may have deleterious consequences for the patient [29].

In a previous study, we demonstrated that autologous transplantation of mice testicular tissue encapsulated in an alginate hydrogel resulted in a 2-fold increase of spermatogonial survival, a result that was suggested to be linked to the antioxidant properties of alginate. The addition of VEGF-loaded nanoparticles (NPs) to reduce the hypoxic period improved short-term angiogenesis but did not further increase spermatogonia survival [30]. 

We hypothesized that localized delivery of a necrosis inhibitor (NECINH) could be an alternative to improve testicular tissue graft outcomes. NecroX-5^TM^ was selected as a candidate molecule as it belongs to the family of indole derivative exerting anti-necrotic properties by scavenging mitochondrial reactive oxygen and nitrogen species [31]. As its anti-necrotic properties in the context of hypoxia/reoxygenation conditions were demonstrated in experiments on rodent hearts [32], we assumed that it could reduce the damages induced in testicular tissue fragments exposed to hypoxia-reoxygenation during the avascular transplantation procedure. We encapsulated NecroX-5 in poly(D,L-lactide-co-glycolide)/poly(ethylene glycol (PLGA/PEG) NPs in order to increase its solubility and to achieve a controlled, sustained, and localized release after its incorporation in a 1% alginate tissue embedding matrix. We evaluated the encapsulation efficiency and release profile of the NPs formulation, and the impact of NECINH supplementation was assessed both in vitro on mouse testicular tissue exposed for 21 days to hypoxia and in vivo after testicular tissue autotransplantation for 5 and 21 days. 

## 2. Results

### 2.1. Characterization of Nanoparticles

NECINH NPs presented a size of 293 +/– 41 nm, a polydispersity index (PDI) of 0.19 +/–0.12, and zeta potential of –39 +/– 6 mV.

**Figure 1:**
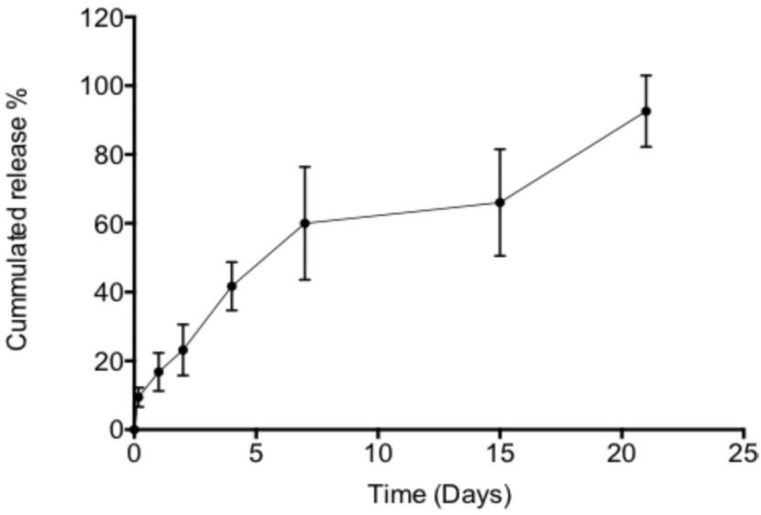
In vitro release of NECINH-nanoparticles (NPs) incorporated in 1% alginate hydrogel. Twenty-five microliters of NPs corresponding to 15.27 µg of NECINH were incorporated in a 50 µl alginate hydrogel and immersed in 40 mM CaCl_2_ PBS (Phosphate buffer saline) for 21 days at 37 °C. The release was evaluated over time by fluorimetry (*n* = 4).

After 7 days of incubation, 64% of encapsulated NECINH was released without significant burst effect, and after 21days, the drug release was almost completed (Figure 1).

### 2.2. Impact of Nanoparticles Containing Necrosis Inhibitor Loaded in Alginate Hydrogel on Testicular Fragments Exposed to Hypoxia In Vitro

**Figure 2:**
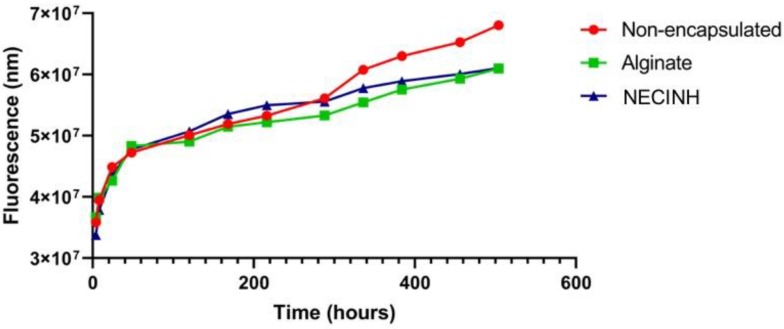
Impact of testicular fragment incorporation in NECINH nanoparticles-loaded alginate hydrogel on tissue necrosis when cultured in hypoxia. Cumulative values of fluorescence corresponding to the lactate dehydrogenase (LDH) measured in culture supernatants recovered every 48–72 h.

Validation of the impact of hypoxia on lactate dehydrogenase (LDH) concentration measured in tissue culture supernatants is presented in the Supplementary Material (Figure S1). LDH concentration increased over time in testicular fragment culture medium in hypoxia, but no significant impact of supplementation with NECINH-NPs or encapsulation in alginate alone was observed (Figure 2).

We analyzed 2484 seminiferous tubule sections (STs), of which 1371 at 5 days (437 for non-encapsulated, 371 for encapsulation in alginate, and 563 for encapsulation in alginate with NPs) and 1113 STs at 21 days (314 for non-encapsulated, 267 for encapsulation in alginate, and 532 for encapsulation in alginate with NPs). Table 1 shows tissue integrity scores in the different groups of grafts. No STs were scored as intact (score 1). At 5 days, the majority of STs appeared satisfactory (Score 2) (89% versus 11% damaged (Score 3)) (Table 1). 

**Figure 3:**
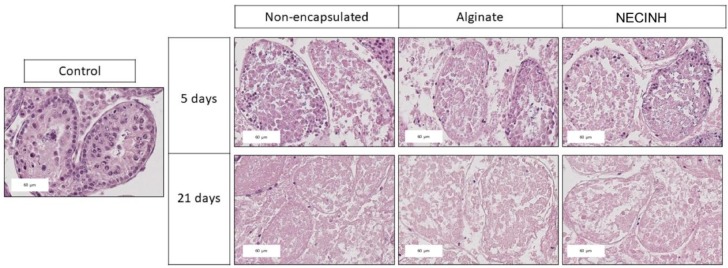
Impact of NECINH-nanoparticles-loaded hydrogel on mice testicular tissue exposed to hypoxia (1% O_2_) and recovered after 5 or 21 days. Three conditions were compared for each timing (non-encapsulated, encapsulated in alginate, and encapsulated in alginate with NECINH NPs). Hematoxylin-eosin staining was used for the evaluation of STs (seminiferous tubule sections) morphology. Scale bar = 60 μm.

At 21 days, this trend was reversed, showing a majority of seminiferous tubules sections presenting a score of 3 over a score of 2 (80% versus 20%). No statistically significant difference was observed between the three groups (Figure 3).

**Figure 4:**
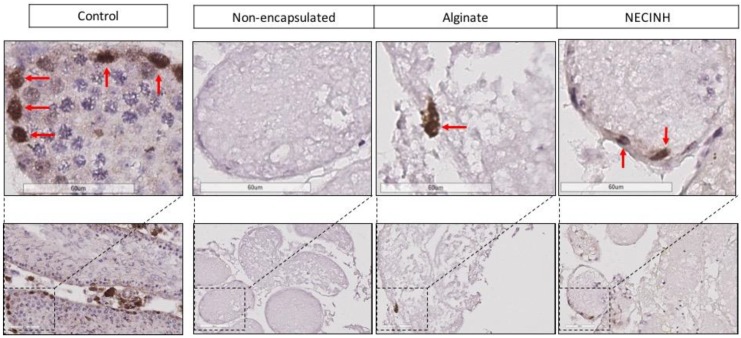
Impact of NECINH-nanoparticles-loaded hydrogel on mice testicular tissue exposed to hypoxia (1% O_2_) for 21 days. Undifferentiated spermatogonial survival. Promyelocytic leukemia zinc finger (PLZF) + cells are highlighted by red arrows.

No undifferentiated spermatogonia identified by promyelocytic leukemia zinc finger (PLZF) staining was found in non-encapsulated tissue fragments after 21 days of culture, while rare PLZF-positive cells were observed in encapsulated tissues, whether it was supplemented with NECINH or not (Figure 4).

### 2.3. Impact of NECINH-Nanoparticles-Loaded Alginate Hydrogel on Testicular Fragment In Vivo Viability

#### 2.3.1. Tissue Integrity 

**Figure 5:**
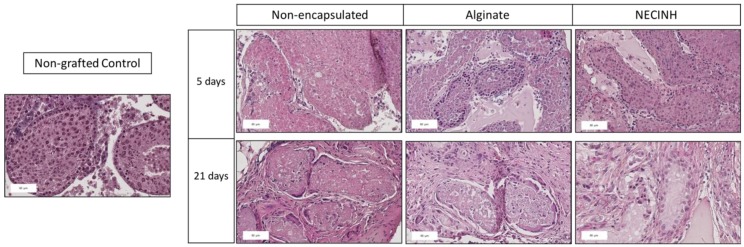
Impact of NECINH-nanoparticles-(NPs) loaded hydrogel on mice testicular tissue after autotransplantation for 5 and 21 days. Hematoxylin-eosin staining was used for the morphological evaluation of seminiferous tubules sections. Scale bar = 60 μm.

For the evaluation of tubular integrity and morphology of recovered tissue on hematoxylin-eosin stained slides (Figure 5), 2157 ST sections were analyzed after 5 days (529 for non-encapsulated tissue, 897 for encapsulation in alginate, and 731 for encapsulation in alginate loaded with NECINH NPs) and 2235 after 21 days (886 for non-encapsulated tissue, 420 for encapsulation in alginate, and 929 for encapsulation in alginate loaded with NECINH NPs). 

Results for each timing and condition are summarized in Table 2.

For each of the three groups, we found an improvement in the number of STs classified as intact and satisfactory (Score 1 + 2) between 5 and 21 days (non-encapsulated tissue: *p* = 0.02; Alginate: *p* = 0.03; NECINH: *p* = 0.01).

**Figure 6:**
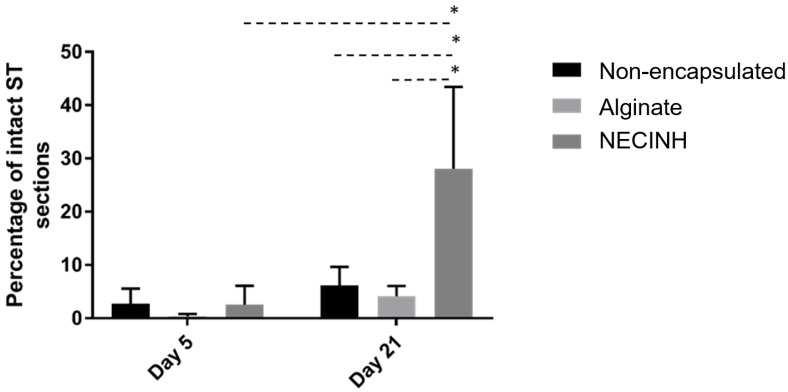
Impact of NECINH-nanoparticles-loaded hydrogel on mice testicular tissue after autotransplantation for 5 and 21 days. Tissue integrity evaluation on hematoxylin-eosin stained slides. The graph shows the percentage of intact (Score 1) STs (seminiferous tubule sections) evaluated at day 5 and day 21. * indicates *p* < 0.05 relative to day 21 Score 1 NECINH-NPs.

After 21 days of transplantation, supplementation of the alginate hydrogel with NECINH-NPs was associated with an increased number of intact STs compared to both non-encapsulated tissue (*p*= 0.007) and tissue encapsulated in alginate (*p* = 0.004). An increase of the STs scored as intact was observed between 5 and 21 days only when testicular tissue was encapsulated in NECINH NPs-loaded hydrogels (*p* < 0.0001) (Figure 6).

#### 2.3.2. Germ Cells’ Survival 

Germ cells’ survival was analyzed on tissue recovered after 21 days of grafting. Immunohistochemistry (IHC) for PLZF identified spermatogonia that did not enter the differentiating cycle, representing a specific germ cell population with the ability to proceed through mitosis or to provide a more differentiated germ cells. In order to evaluate more differentiated germ cells, such as spermatocytes, we performed IHC for protein boule-like (BOLL). VASA was used as a second marker for both spermatogonia and spermatocytes. 

**Figure 7:**
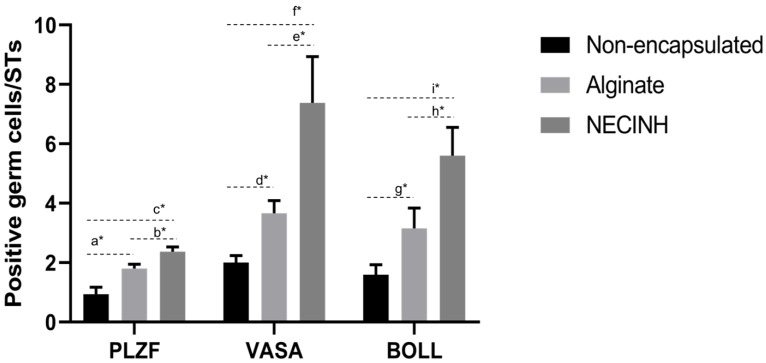
Impact of NECINH-nanoparticles (NPs)-loaded hydrogel on germ cells survival in mice testicular tissue after 21 days of autotransplantation. The number of positive cells/ST section for each germ cell marker (Promyelocytic leukemia zinc finger (PLZF), VASA, and protein boule-like (BOLL)) was statistically significantly higher for NECINH encapsulation compared to both alginate-only and non-encapsulation and for alginate-only encapsulation compared to non-encapsulation. Results are expressed as mean number of positive cells/seminiferous tubule section ± standard deviation. For PLZF, Non-encapsulated: 0.94 ± 0.23; Alginate: 1.80 ± 0.14; NECINH 2.37 ± 0.15. a: alginate/non-encapsulated tissue (*p* < 0.0001); b: NECINH NPs/non-encapsulated tissue (*p* < 0.0001); c: NECINH NPs/alginate (*p* = 0.0009). For VASA, Non-encapsulated: 2 ± 0.23; Alginate: 3.66 ± 0.43; NECINH 7.38 ± 1.56 d: alginate/non-encapsulated tissue (*p* = 0.0415); e: NECINH-NPs/ alginate (*p* = 0.0001); f: NECINH-NPs/non-encapsulated tissue (*p* < 0.0001). For BOLL, Non-encapsulated: 1.60 ± 0.34; Alginate: 3.16 ± 0.68; NECINH 5.60 ± 0.95. g: alginate/non-encapsulated tissue (*p* = 0.00111); h: NECINH-NPs/alginate (*p* = 0.0004); i: NECINH-NPs/non-encapsulated tissue (*p* < 0.0001). * statistical significant results (*p* < 0.05).

**Figure 8:**
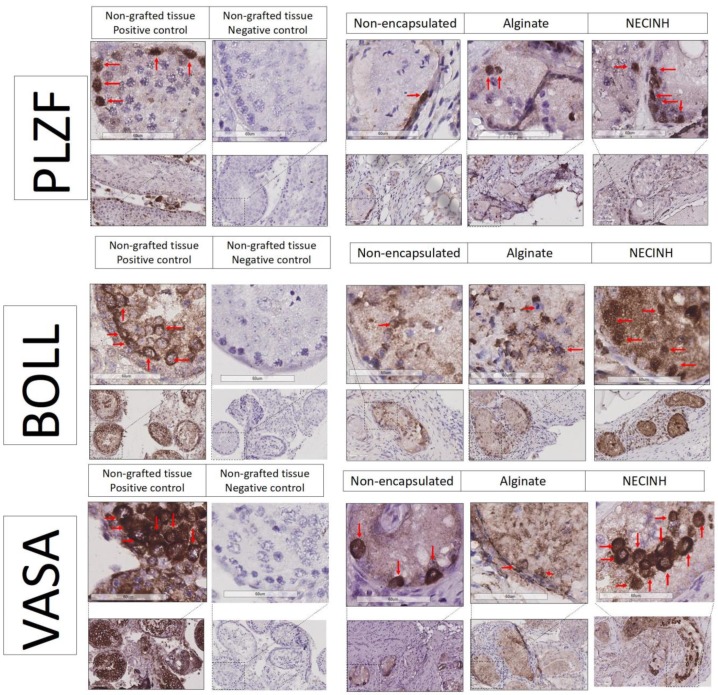
Impact of NECINH-NPs on germ cells survival in mice testicular tissue auto-transplanted for 21 days. Images show 40 X magnification of IHC for PLZF, BOLL, and VASA. Positive cells are highlighted by red arrows.

Significantly higher numbers of germ cells survived when the testicular tissue was encapsulated in NECINH NPs-loaded alginate hydrogels. IHC for PLZF, BOLL, and VASA showed a significantly higher number of positive cells/seminiferous tubule sections when tissue encapsulated with NECINH-NPs was compared to both non-encapsulated tissue and tissue encapsulated in alginate, and when the tissue was encapsulated in alginate compared to non-encapsulated tissue (Figure 7 and Figure 8). 

#### 2.3.3. Intratubular Apoptosis 

Active caspase-3 was used as the end-marker of the caspase-dependent apoptotic cell death pathway to identify cells going through apoptosis.

No statistical difference between the three conditions was found regarding the number of active caspase-3 positive cells/STs after 5 or after 21 days (Table 3). see Figure S2.

#### 2.3.4. Oxidative Stress Evaluation Using Malondialdehyde (MDA)

As exposure to oxidative stress induces the peroxidation of cell membranes’ polyunsaturated fatty acids, cells accumulating MDA as an end-product of lipid oxidation were identified to assess the protective effect of NECINH-NPs against oxidative stress in tissue grafts. 

**Figure 9:**
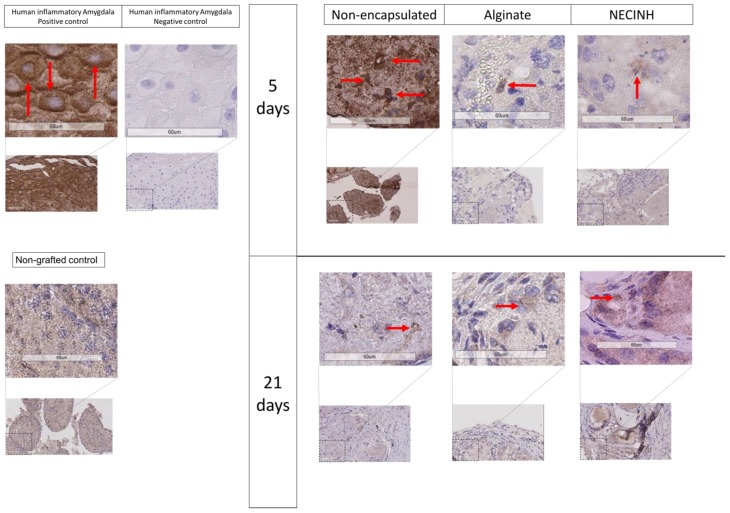
Impact of NECINH nanoparticles (NPs)-loaded hydrogel on lipid peroxidation in mice testicular tissue after autotransplantation for 5 and 21 days. Malondialdehyde (MDA)-positive cells were reduced on day 5 when encapsulation in alginate was performed, regardless of NPs supplementation. Positive cells are highlighted by red arrows.

Five days after transplantation, significantly more MDA-positive cells were observed for non-encapsulated tissue compared to both tissue encapsulated in alginate (*p* = 0.0073) and alginate with NECINH-NPs (*p* = 0.0132) (Table 3, Figure 9). After 21 days of grafting, no difference between the three conditions was found (Table 3, Figure 9).

## 3. Discussion

The results of our study represented a significant step towards the development of optimal transplantation procedures that are awaited before considering autotransplantation of cryobanked ITT from prepubertal boys aiming at future fertility restoration.

Indeed, encapsulation in a 1% alginate matrix with supplementation of NPs allowing localized, controlled, and sustained delivery of necrosis inhibitor improved spermatogonial and germ cells’ survival and tissue integrity in mouse testicular tissue avascular autografts, a model also previously used to assess human ITT xenografts where an important spermatogonial loss was observed [17,33,34]. 

All strategies that are intended to restore a patient’s fertility using his own frozen-thawed ITT, using in vivo or in vitro approaches rely on the presence of spermatogonia as precursors of spermatozoa [4]. Furthermore, in both mouse and human testicles, spermatogonial stem cells have been quantified as scarce [27,35], and their number seems to be further reduced in patients affected by diseases, such as sickle cell anemia and leukemia [2]. 

Hence, as far as it concerns cryostored ITT, increasing spermatogonial survival represents a challenge, whatever the technique that could offer an option for fertility restoration [36].

The objective of this study was to evaluate a new approach to increase the transplantation efficiency by reducing oxidative stress based on well-known antioxidant properties of alginate [37] and by diminishing tissue necrosis with a necrosis inhibitor. To evaluate the impact of our combinational approach on hypoxia-reoxygenation events in grafts, we decided to evaluate tissue integrity after 5 days of transplantation to study the effect of the hypoxia that precedes the revascularization, and after 21 days to investigate the effect of revascularization and reoxygenation. 

Previous experiments involving mice testicular tissue autotransplantation showed that NPs delivering VEGF improved short-term vascularization without exerting any effect on spermatogonia survival and that alginate itself, regardless of the addition of VEGF-NPs, increased the survival of spermatogonia [30].

We here showed that the incorporation of testicular tissue in NECINH-NPs-loaded alginate hydrogel significantly improved both tissue integrity and spermatogonial survival when compared to encapsulation in alginate alone, progressing towards better chances of achieving successful fertility restoration with ITT transplantation.

No previous reports are available concerning the delivery of NECINH with NPs, and our results provided insights into a new tool that might be applied to fertility preservation techniques. Nanomedicines loaded with NECINH were successfully prepared, and the obtained, sustained, and controlled release profile was ideal as the first-week post-transplantation was shown to be critical for graft development [21].

Validating the ex vivo model of hypoxia-induced toxicity provided an effective and accessible tool to explore the impact of hypoxia on testicular tissue. This experimental model was used to evaluate the influence of NECINH-NPs-supplemented hydrogel on tissue and cell viability in vitro. The absence of NECINH-NPs toxicity on mice testicular tissue was observed based on LDH quantification. These results were supported by a significant increase of spermatogonia surviving after in vivo transplantation compared to alginate alone as a valuable achievement in the field of fertility preservation. As the mouse spermatogenic cycle lasts for 35 days [38], we expected partial germ cell differentiation in the tissue recovered after 21 days of transplantation, and we also evaluated the presence of spermatocytes, a population of germ cells more differentiated than spermatogonia [39]. The increased numbers of spermatocytes demonstrated by immunostaining for both BOLL and VASA represented a further confirmation of the positive effect of NECINH-NPs on testicular tissue. Besides the enhanced survival of undifferentiated spermatogonia (PLZF-positive cells), we confirmed their short-term ability to enter the differentiation pathway and thus the potential to deliver more differentiated germ cells with NECINH-NPs.

As a reduction in MDA-positive cells/seminiferous tubule was observed 5 days after transplantation, regardless of supplementation with NECINH-NPs, the reduction in membrane lipid peroxidation and thus reduced oxidative stress was most likely due to the antioxidant properties of alginate [40].

Immunostaining for active caspase-3 on tissue recovered after 5 and 21 days of transplantation did not point to any effect of NECINH-NPs supplementation on caspase-dependent apoptosis. The few previous studies investigating possible effects of NECINH NecroX-5^TM^ on apoptosis are controversial. Indeed, NecroX-5^TM^ administration was shown to interfere with apoptosis, causing inhibition of caspase-3 cleavage and downregulation of Bcl-2 protein [41], as well as a reduction of DNA fragmentation evidenced by terminal deoxynucleotidyl transferase dUTP nick end labeling (TUNEL) [42,43]. By contrast, our observations were in agreement with those of Kim et al. [31], who did not see such effect with the administration of a molecule acting on caspase-dependent apoptosis on rat hepatocytes exposed in vitro to tertiary-butyl-hydroperoxide (t-BHP)-induced cellular injury. In addition, besides the anti-necrosis properties of NecroX-5^TM^, anti-inflammatory properties previously described [44], as well as a recently reported effect on macrophage response [45], could have contributed to the results we obtained. A deeper understanding of the pathways involved in the NecroX-5^TM^ mode of action, especially when delivered in such a localized and sustained fashion, would be useful for further applications. Increasing NECINH concentration in the encapsulation matrix beyond the threshold of 10 μM might further improve tissue survival, as a dose-dependent effect was documented for myoblasts exposed in vitro to tert-butyl-hydroperoxide [31], and the half-maximal inhibitory concentration (IC50) tested in vitro was beyond 96 μM [46].

Evaluating transplantation results over a period longer than 21 days (the period needed for neovessels stabilization) [23] might also provide more information concerning germ cell differentiation and functionality in future experiments. However, animal tissue was used in these series of experiments to allow comparison of several groups of treatment in vitro and in vivo using a completely new strategy for the transplantation procedure and thus avoid unethical use of scarce human prepubertal tissue available for research purposes.

Before considering any clinical application of ITT transplantation, several issues concerning the safety of the procedure should still be addressed besides the risk linked to potential contamination of the tissue with cancer cells. Indeed, the possibility of epigenetic modifications that could be induced by the transplantation and tissue encapsulation environment should be evaluated.

With regard to the administration to humans, in spite of the encouraging results obtained in experiments carried out both in vitro and in vivo, the efficacy and safety of NPs should further be addressed [47]. In order to be effective, NPs should allow localized interaction of the drug with the target tissue and cells, thus avoid reticuloendothelial system sequestration and phagocytosis by macrophages and at the same time increase stability and solubility of the encapsulated molecules [48]. Pharmacology interventions aimed at reaching these objectives are supposed to take into consideration possible adverse effects, such as potential toxicity of the products of degradation of the polymer used for encapsulation and the accumulation and diffusion of NPs to the central nervous system after passing the blood-brain barrier [49]. Three-dimensional images of nanoparticles obtained through techniques like scanning electron microscopy (SEM) [50] could help to provide further insights on factors that could interfere with drug delivery.

Moreover, while alginate [51] and PLGA nanomedicines [52] have been authorized by the food and drug administration for use in humans, this does not apply to molecules belonging to the NecroX family yet.

## 4. Materials and Methods

### 4.1. NECINH Encapsulation in PLGA:PLGA-PEG Nanoparticles

PLAG-PEG NPs containing NECINH were produced by single emulsion [53] and characterized in terms of morphology, zeta potential, size, and encapsulation efficiency.

Thirty-five milligrams of PLGA (Resomer^®^ RG 502H, Sigma-Aldrich-719897, Darmstadt, Germany) and 15 mg of PLGA-PEG (Resomer^®^ RGP d 50155, Boehringer Ingelheim, Ingelheim, Germany) were dissolved in 1 mL dichloromethane (CH_2_CL_2_). Two hundred fifty micrograms of NECINH NecroX-5^TM^ in acetone and 2 mL of 1% poly (vinyl alcohol) (PVA) (88% Hydrolyzed, MW 22000-30000, Across Organic 396765000) were added to the solution. An emulsion was obtained by sonication (70W, 15 s) that was then added drop by drop onto 100 mL of 0.3% PVA solution and stirred (room temperature, 550 rpm) to allow the solvent to evaporate. Hardened NPs were then centrifuged at 22,000× *g* for 30 min at 4 °C and washed 3 times in water. NPs were then resuspended in 1 mL Milli-Q water (stock solution).

#### 4.1.1. Physico-Chemical Characterization of NecroX-5 Nanoparticles

Size, zeta-potential, and PDI (polydispersity index) of NecroX-5 NPs were measured using a Zetasizer (Malvern Panalytical, Malvern, UK). Samples were diluted 1/100 (v/v) in Milli-Q water, and the encapsulation efficiency of NecroX-5 was calculated by using the following formula:

$$Encapsulation\;Efficiency=\frac{NecroX-5\;introduced\;in\;the\;formulation-encapsulated\;NecroX-5}{NecroX-5\;introduced\;in\;the\;formulation}*100$$

Necrox-5 concentration was measured by fluorimetry (excitation wave λ = 330 nm, emission wave λ = 460 nm).

#### 4.1.2. NecroX-5 In Vitro Release

NecroX-5 NPs were dispersed in 45 µL of 1% alginate (SLM100, FMC BioPolymers, NovaMatrix™, Sandvika, Norway) solution in 3-(*n*-morpholino)propanesulfonic acid, 4-morpholinepropanesulfonic acid (MOPS buffer) (M3183; Sigma-Aldrich, Darmstadt, Germany), prepared as previously described [30], and 5 µL of CaCl_2_ (50 mM solution in MOPS) was added to initiate gelation.

Hydrogels were placed in 96-well plates, covered with 250 µL of 40 mM CaCl_2_ PBS, and incubated at 37 °C. Release media were collected at different timings (4 h, and 1, 2, 4, 10, 15, 21 days). Samples were centrifuged, and NecroX-5 was quantified by fluorimetry after a 2-times dilution in PBS. Results were expressed as the percentage of released NecroX-5 compared to the total amount of NecroX-5 added in the hydrogel.

### 4.2. Ethical Approval and Animal Care

All experiments involving animals were approved by the Ethics Review Board and the Committee on Animal Research of UCLouvain (project 2018/UCL/MD/20, approval date: 22 June 2018) according to the laws currently in force in Belgium (Royal Decree on the Protection of Experimental Animals 29th Mai, 2013). NMRI (Naval Medical Research Institute) mice were purchased directly from the animal facility of UCLouvain and were kept in cages with usual night/day cycle with water and food available ad libitum and received the animal care in compliance with university guidelines. Animals were randomly assigned to experimental or control groups (5 mice per group). All experimental surgical procedures were carried out inside the university animal housing facility (Linné building, UCLouvain, Woluwe Saint-Lambert, Belgium), and no adverse reaction was documented.

### 4.3. Impact of Nanoparticles Containing Necrosis Inhibitor on Tissue Necrosis In Vitro

To validate that incubation of testicular tissue fragments in hypoxia-induced cytolysis, lactate dehydrogenase (LDH) levels in the supernatant of tissues kept for 5 days in hypoxia (incubator with 1% O_2_) (INVIVO2400 hypoxic chamber, Ruskinn Technology, Ltd., Bridgend UK) or in normoxia (21% O_2_) were first compared (Figure S1).

Testicles were collected after bilateral orchidectomy, as previously described, from 3 NMRI mice aged between 4 and 5 weeks and weighted 28–32 g. The testicular tissue obtained from both testicles was cut in small fragments (±1 mm^3^). One percent of alginate hydrogel was used for tissue embedding to facilitate manipulations [30]. Tissue fragments were placed in a sterile petri dish containing 45 μL of 1% alginate solution and 10 µM of NECINH-NPs with the further addition of 5 μL of CaCl_2_ to initiate the gelation. Non-encapsulated tissue fragments and fragments encapsulated in alginate alone were used as negative controls. Four fragments per mouse (2 of each testis) were cultured for each of the three groups. All fragments were placed on Millicell^®^ (Millipore, cell culture inserts, 12 mm/0.4 μm, Darmstadt, Germany) on 24-well plates keeping an air-liquid interface [54]. Three hundred microliters of culture medium composed of α-MEM (α-minimum essential medium, without phenol Gibco^TM^, 32561029, Waltham, MA, USA), Penicillin/Streptomycin (Thermo-Fisher^TM^, 15070063, Waltham, MA, USA), and FBS (fetal bovine serum) (ThermoFisher^TM^, 10500056, Waltham, MA, USA) were added per well. Samples were kept in a hypoxia incubator (INVIVO2400 hypoxic chamber, Ruskinn Technology, Ltd., Bridgend UK) set at 1% of O_2_ for up to 21 days. One testicular tissue fragment per mouse was collected after 5 days and 3 after 21 days for histological and immunohistochemical analyses (see Section 4.5). One testicular fragment per mouse was also fixed immediately after sampling in mDF (modified Davidson’s fluid) as a positive control. Culture media were collected after 4, 8, 24 h, and then every 48–72 h up to 21 days and stored at −20 °C until further analysis. Cytotoxicity was evaluated by measuring LDH concentration according to the supplier instructions (CytoTOX-ONE kit (PromegaTM G7890, Leiden, Netherlands)).

### 4.4. Impact of Nanoparticles Containing Necrosis Inhibitor In Vivo on Auto-Transplanted Testicular Tissue

NMRI mice (*n*= 30) were anesthetized by an intraperitoneal injection of medetomidine (1 mg/kg) (Domitor, Pfizer, Cambridge, MA, USA) and ketamine (75 mg/kg) (Anesketin, Eurovet, Heusden-Zolder, Belgium). Analgesia was provided by intraperitoneal injection of buprenorphine (0.1 mg/kg) (Temgesic, Schering Plough, Kenilworth, NJ, USA). Intraperitoneal injection of atipamezole (1 mg/kg) (Antisedan, Pfizer, Cambridge, MA, USA) was used to reverse anesthesia.

After bilateral orchidectomy, one ±1 mm^3^ fragment of autologous testicular tissue was grafted into the scrotum, as previously described [33], without the creation of an artificial vascular anastomosis. Fragments were encapsulated, as described in Section 4.3 Fragments encapsulated in NECINH NPs-loaded hydrogels were compared to controls (non-encapsulated and encapsulated in alginate alone) (*n* = 5/group). Mice were euthanized by cervical dislocation after 5 or 21 days. Grafts were recovered and fixed in mDF, embedded in paraffin, and cut into 5 μm-thick serial sections that were placed per pair on Superfrost Plus slides.

### 4.5. Histology and Immunohistochemistry Analyses

For immunohistochemistry, tissue sections were deparaffinized according to common protocols. To block endogenous peroxidase activity, slides were incubated for 30 min at room temperature (RT) in 0.3% H_2_O_2_. After washing in distilled water, slides were immersed in citrate buffer for 60 min at 98 °C for antigen retrieval and incubated for 30 min at RT with 10% normal goat serum (NGS, Invitrogen, Merelbeke, Belgium) and 1% bovine serum albumin (BSA) (Invitrogen, Merelbeke, Belgium) to block non-specific binding sites. Primary antibodies were added to the slides according to the manufacturer’s suggested concentrations and incubated at 4 °C. We used anti-promyelocytic leukemia zinc finger (PLZF) (rabbit anti-PLZF antibody, 1/400, Sigma-Aldrich, HPA001499, St. Louis, MO, USA) as a marker of undifferentiated spermatogonia [55]; VASA (rabbit anti-VASA antibody, 1/2000, ab13840, Abcam, Cambridge, UK) as a marker of spermatogonia, spermatocytes, and rounds spermatids [56]; BOLL (rabbit anti-BOLL antibody, 1/1600, Sigma-Aldrich, HPA 0488-13, St. Louis, MO, USA) as a marker of spermatocytes and round spermatids [57]; anti-active caspase 3 (rabbit anti active caspase-3, 1/200, Promega G7481, Leiden, Netherlands) to identify cells expressing cleaved caspase 3 [58]; and anti-malondialdehyde (MDA) (rabbit anti-MDA antibody, 1/1000, Ab6463, Abcam, Cambridge, UK) to identify cells presenting evidence of lipid peroxidation as a sign of oxidative stress [59,60]. A secondary anti-rabbit antibody (Envision+ system-labeled polymer-horseradish peroxidase (HRP); DAKO, K4003) was added to the sections and incubated for 60 min at RT. Diaminobenzidine (DAKO K3468) was used as chromogen, and Mayer’s hematoxylin was employed to counterstain nuclei. After washing with tap water, slides were dehydrated with serial baths in methanol and toluene, and Entellan mounting medium (Sigma-Aldrich, St. Louis, MO, USA) was used with coverslips to seal the slides. All tissues were analyzed on digital images captured with a Leica SCN400 slide scanner (Leica Biosystems, WETZLAP, Nußloch Germany).

Results were expressed as the mean number of positive cells per seminiferous tubule (ST) section. For the ST integrity score, a slide every 50 μm was stained with hematoxylin-eosin (HE). We used the scoring criteria previously described [30] to assess ST integrity (Figure S3).

### 4.6. Statistical Analysis

Statistical analyses were performed using GraphPad Prism (GraphPad Software, La Jolla, CA, USA).

Shapiro-Wilk test was applied to validate the normal distribution of data, and one-way ANOVA with Tukey posthoc test was used to compare the means of three groups. Results are expressed as mean +/– standard deviation and were considered significant if *p* < 0.05.

## 5. Conclusion

Our results confirm that our strategy has a relevant potential for further studies involving human tissue. The testicular tissue transplantation experiments with alginate hydrogel and VEGF NPs [30] and here with NECINH-NPs represent a paradigm for future applications of tissue engineering procedures applied to fertility preservation and other fields of regenerative medicine.

## Figures and Tables

**Table 1:** Impact of NECINH-nanoparticles (NPs)-loaded hydrogel on mice testicular tissue exposed to hypoxia (1% O_2_) for 5 and 21 days. Results for tissue integrity are expressed as the mean percentage of seminiferous tubules for each integrity score and condition ± standard deviation.

5 Days	Non-Encapsulated	Alginate	NECINH-NPs
Score 2	93% ± 5%	87% ± 14%	88% ± 8%
Score 3	6% ± 5%	13% ± 14%	11% ± 8%
**21 Days**	**Non-Encapsulated**	**Alginate**	**NECINH-NPs**
Score 2	18% ± 11%	19% ± 7	20% ± 1 %
Score 3	80% ± 12	80% ± 7	78 ± 0.6%

**Table 2:** Impact of NECINH-nanoparticles(NPs)-loaded hydrogel on mice testicular tissue after autotransplantation for 5 and 21 days. Results for tissue integrity are expressed as the mean percentage of STs (seminiferous tubule sections) for each integrity score and condition ± standard deviation. *n* = 30. * indicates *p* < 0.05 relative to day 21 Score 1 NECINH-NPs.

5 Days	Integrity Score of STs		
	Non-Encapsulated	Alginate	NECINH-NPs
Score 1	2.7% ± 3%	0.35% ± 0%	2.58% ± 4% *
Score 2	37% ± 27%	31.7% ± 15%	45.8% ± 18%
Score 3	60% ± 26%	69.1% ± 13%	51.6% ± 18%
**21 days**	**Integrity score of STs**		
	**Non-encapsulated**	**Alginate**	**NECINH-NPs**
Score 1	5% ± 3% *	4.1% ± 1.9% *	28% ± 15%
Score 2	65% ± 14%	59% ± 23%	56% ± 6%
Score 3	29% ± 13%	37% ± 24%	18% ± 15%

**Table 3:** Impact of NECINH-nanoparticles(NPs)-loaded hydrogel on apoptosis (active caspase 3-positive cells) and membrane lipid peroxidation (Malondialdehyde (MDA)-positive cells). After 5 days of transplantation, no effect on apoptosis was observed. MDA+ cells were reduced in alginate and NECINH groups. After 21 days of transplantation, no effect on apoptosis or lipid peroxidation was observed. Results are expressed as a mean number of positive cells/seminiferous tubule section ± standard deviation. * indicates *p* < 0.05 relative to day 5 non-encapsulated.

5 days	Non-Encapsulated	Alginate	NECINH-NPs
Active-caspase 3	2.7 ± 1	2.7 ± 0.7	1.9 ± 0.5
MDA	1.3 ± 0.9	0.09 ± 0.09 *	0.2 ± 0.2 *
**21 days**	**Non-Encapsulated**	**Alginate**	**NECINH-NPs**
Active-caspase 3	1.3 ± 0.5	1.4 ± 0.5	0.9 ± 0.5
MDA	0.9 ± 0.2	1.4 ± 1.5	0.6 ± 0.2

## Supplementary Materials

Supplementary materials can be found at https://www.mdpi.com/1422-0067/20/23/5833/s1. Figure S1. Validation experience. Figure S2. Impact of NECINH nanoparticles(NPs) loaded hydrogel on apoptosis in mice testicular tissue after auto-transplantation for 5 and 21 days. Figure S3. Seminiferous tubules sections scoring criteria.
