# Supplementary material for: Significant Benefits of Nanoparticles Containing a Necrosis Inhibitor on Mice Testicular Tissue Autografts Outcomes
Source: Int J Mol Sci. 2019 Nov 20;20(23):5833. doi: 10.3390/ijms20235833 (PMC6929043; doi:10.3390/ijms20235833)
Supplement: Supplementary file 1 [file ijms-20-05833-s001.pdf]

Supplementary material

I. Validation of the method for tissue cytotoxicity assessment:

A preliminary experiment was performed in order to validate the culture in hypoxia incubator with 1% O_2 as a method to induce cytotoxicity. Two parallel in vitro culture experiments for 5 days using 1 mm^3 fragments of testicular tissue obtained from three different NMRI (Naval Medical Research Institute) mice (Faculty UCL, Linné, Brussels, Belgium) aged between 4 and 5 weeks were conducted. For each mouse, 5 testicular tissue fragments were cultured either in an incubator with 21% O_2, or in an incubator in hypoxia condition with 1% 0_2.

Tissue fragments were placed on Millicell inserts inside wells of a 24-well plate (Millipore—CM cell culture inserts, 12 mm/0.4 µm, Merck, PICM01250) with an air-liquid interface and 300 µL of culture medium composed of α-MEM (α-minimum essential medium, without phenol Gibco™ 32561029), 1% Penicilline/Streptomicine (Thermo-Fisher™15070063) and 10 % FBS (fetal bovine serum) (ThermoFisher™ 10500056). Supernatants were collected after 4, 8, 24, 48 and 96 h, stored at −20 °C until analysis and used for determination of LDH (lactate dehydrogenase) by fluorimetry (CytoTOX-ONE kit, Promega™ G7890). Reagent were prepared according to the manufacturer's indications, and the average fluorescence value of the culture medium background was subtracted from all fluorescence values of experimental wells. Experience was repeated twice.

II. Validation experience results

Fluorometric determination of LDH in supernatants of cultured tissue fragments at every timepoint is shown in Figure 5. Using linear mixed models, results for hypoxia were found to be significantly higher than those for normoxia ($p = 0.03$) when all the data were taken into account (overall effect) but also when each timepoint was analyzed separately (5 separate analyses were done for each timepoint 4, 8, 24, 48, 96 h) (Figure S2).

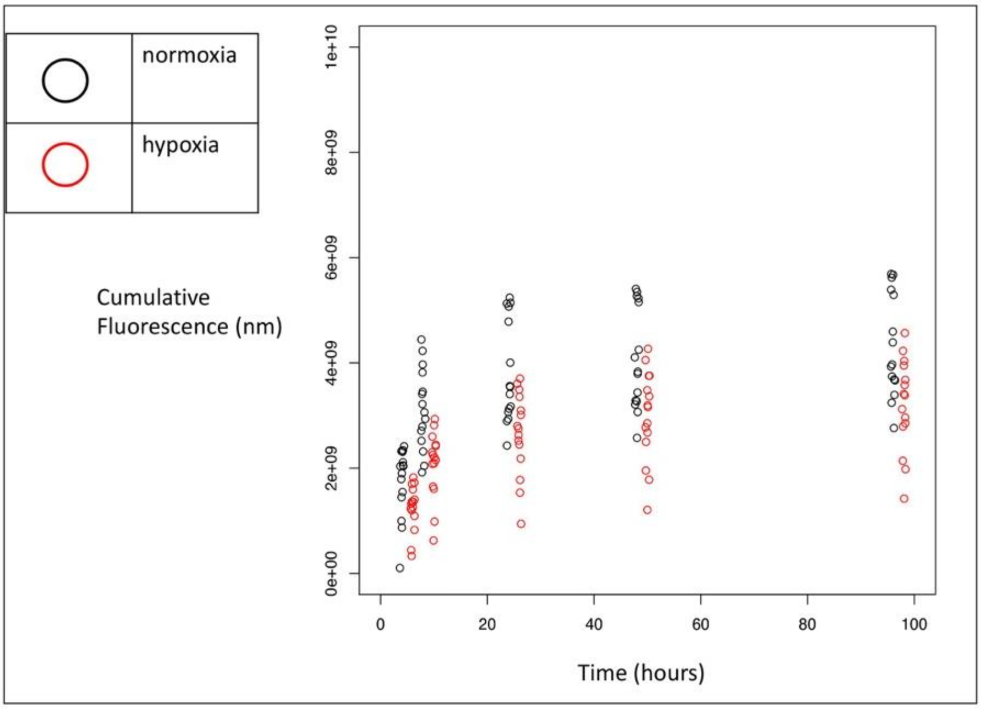

Figure S1. validation experience, parallel culture in hypoxia incubator (1% O_2) and normoxia (21% O_2). Fluorescence in nanometers (nm) corresponding to the LDH (lactate dehydrogenase) concentration in culture medium was statistically significant higher in the hypoxic condition ($p < 0.05$).

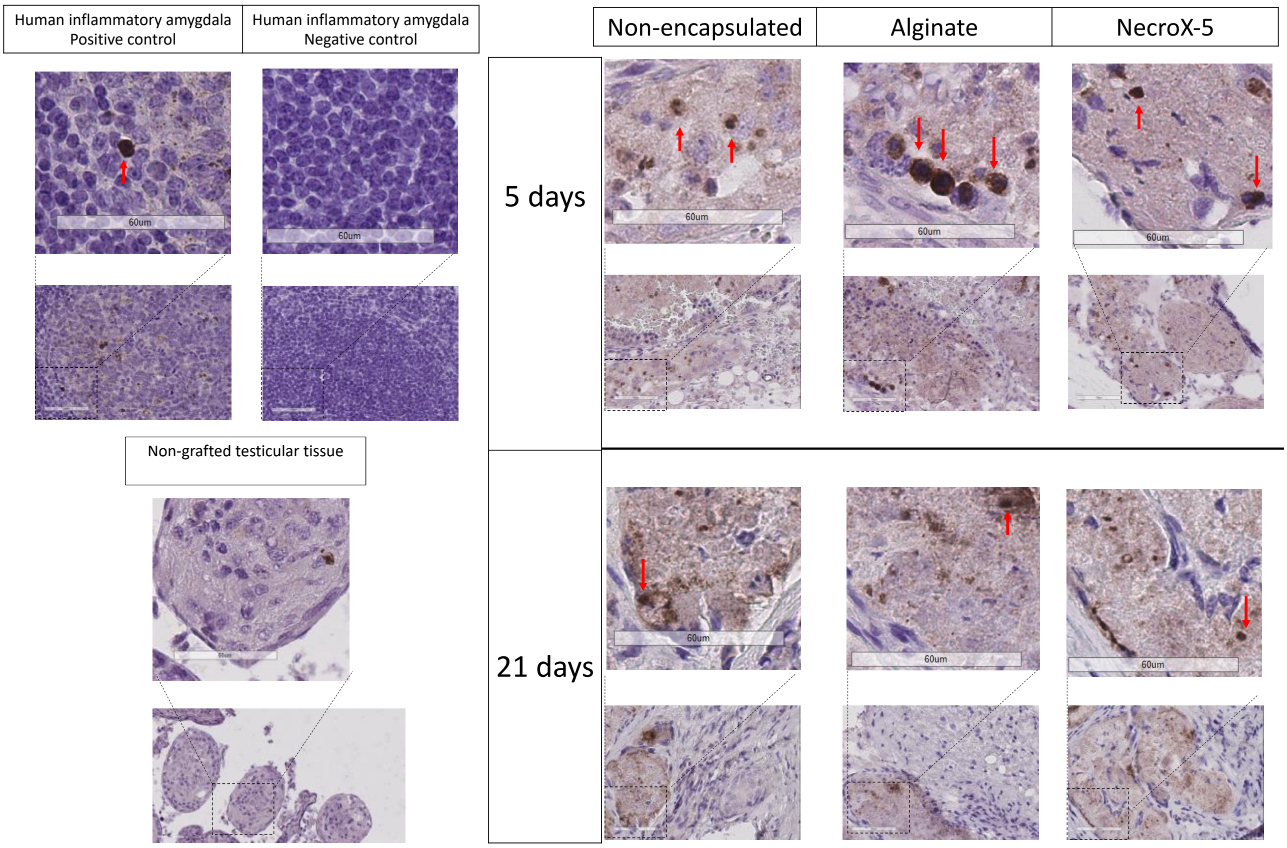

Figure S2. Impact of NECININH nanoparticles(NPs) loaded hydrogel on apoptosis in mice testicular tissue after auto-transplantation for 5 and 21 days. Images show Active Caspase-3 IHC. Positive cells are highlighted by red arrows.

	**Parameters (5 and 21 days)**		**Non-transplanted control**
**Intact** (score 1)	• cells adhered to the basement membrane<br>• there was cohesion between cells<br>• no necrosis was noted	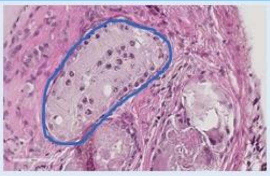	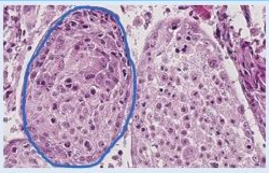
**Satisfactory** (score 2)	• intratubular cells could still be individualized despite the presence of focal necrosis	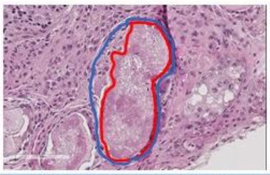	
**Damaged** (Score 3)	• complete necrosis was observed	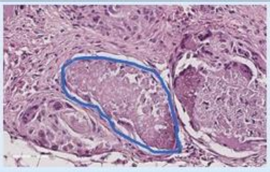	

Figure S3. Seminiferous tubules sections scoring criteria. Blue lines highlight seminiferous tubules (ST). Tubules were classified intact when (i) cells were adherent to the basement membrane, (ii) there was cohesion between cells, and (iii) no necrosis was spotted (score 1). Tubules were classified satisfactory when in spite of the presence of focal necrosis, intratubular cells could still be individualized (score 2). The red line highlights focal necrosis. Tubules were classified damaged when we observed complete necrosis (score 3).
